# S1 represents multisensory contexts and somatotopic locations within and outside the bounds of the cortical homunculus

**DOI:** 10.1016/j.celrep.2023.112312

**Published:** 2023-03-30

**Authors:** Isabelle A. Rosenthal, Luke Bashford, Spencer Kellis, Kelsie Pejsa, Brian Lee, Charles Liu, Richard A. Andersen

**Affiliations:** 1 Biology and Biological Engineering, California Institute of Technology, Pasadena, CA 91125, USA; 2 T&C Chen Brain-machine Interface Center, California Institute of Technology, Pasadena, CA 91125, USA; 3 Department of Neurological Surgery, Keck School of Medicine of USC, Los Angeles, CA 90033, USA; 4 USC Neurorestoration Center, Keck School of Medicine of USC, Los Angeles, CA 90033, USA; 5 Rancho Los Amigos National Rehabilitation Center, Downey, CA 90242, USA; 6 Lead contact

## Abstract

Recent literature suggests that tactile events are represented in the primary somatosensory cortex (S1) beyond its long-established topography; in addition, the extent to which S1 is modulated by vision remains unclear. To better characterize S1, human electrophysiological data were recorded during touches to the forearm or finger. Conditions included visually observed physical touches, physical touches without vision, and visual touches without physical contact. Two major findings emerge from this dataset. First, vision strongly modulates S1 area 1, but only if there is a physical element to the touch, suggesting that passive touch observation is insufficient to elicit neural responses. Second, despite recording in a putative arm area of S1, neural activity represents both arm and finger stimuli during physical touches. Arm touches are encoded more strongly and specifically, supporting the idea that S1 encodes tactile events primarily through its topographic organization but also more generally, encompassing other areas of the body.

## In brief

Rosenthal et al. examine the arm region of the human primary somatosensory cortex and show that it is modulated by vision during physical touches but is unresponsive during passive visual observation alone. They also show that this region encodes information from two distinct body locations, despite the area’s classical topographic organization.

## Graphical Abstract

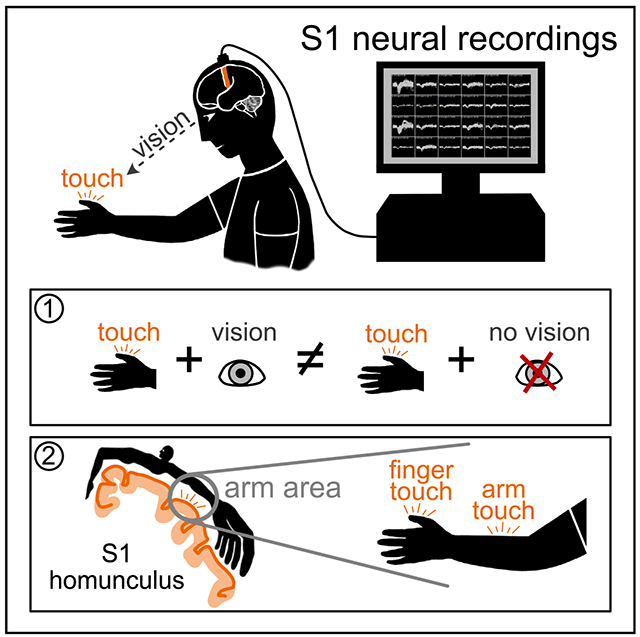

## INTRODUCTION

The sense of touch is important for implementing dexterous, adaptable action plans^1–5^ and creating a sense of ownership and agency over one’s body.^6–8^ The primary source of information for tactile sensations is input from peripheral mechanoreceptors, but multisensory integration^9,10^ plays a role as well, especially visual information.^11–15^

The primary somatosensory cortex (S1) is one of the first cortical areas to receive incoming tactile information, relayed via the cuneate nucleus and the thalamus.^16^ S1’s responsiveness to physical touch and its topographic organization have been extensively documented,^17–21^ but the extent to which multisensory information is represented in S1 remains under investigation. A large body of literature addressing this precise question has found that S1 responds to observed touch when it occurs in others, but not oneself.^22–30^ However, a significant number of studies have failed to find evidence of this phenomenon.^31–34^

A similar but distinct question concerns whether S1 is modulated by vision when it is paired with a physical touch event. Psychophysically, the visual enhancement of touch has been well established: tactile acuity is enhanced when a touched area is observed, even when the visual input is non-informative,^14,35–38^ although the precise conditions necessary to trigger the effect are still unclear.^39^ Electroencephalographic (EEG) experiments have shown that combining visual and tactile stimuli modulates the P50 somatosensory-evoked potential, which is thought to originate in S1.^40–43^ Magnetoencephalography (MEG) studies have suggested that the topographic mapping of fingers shifts in S1 based on the relative timing of visual and tactile signals.^44,45^ Transcranial magnetic stimulation (TMS) over S1 negatively affects the ability to detect or discriminate touches, if the accompanying visual information incorporates a human hand rather than a neutral object.^46–48^ Thus, biologically relevant visual information appears to be used as a predictive signal and modulates S1 encodings of tactile events.

Like its role in integrating multisensory stimuli, S1’s topographic organization appears to be more nuanced than first thought. Recent experiments suggest that although S1 maintains a gross topographic representation of the body as laid out in the earliest human cortical stimulation studies and observed many times since,^17–21,49^ it also contains other more complex levels of tactile representation.^50–52^ Studies of the primate hand have shown that S1 neural activity contains non-linear interactions across different digits,^50,52,53^ supporting the idea that S1 carries information beyond a linear report of inputs from tactile receptors. In humans, S1 has recently been shown to represent body parts outside of their traditionally defined areas.^54,55^

To interrogate S1’s representations of touch across body locations and multisensory contexts, electrophysiological recordings in a human tetraplegic patient with two microelectrode arrays (Blackrock Neurotech, Salt Lake City, UT, USA) implanted in the putative area 1 of the S1 arm region^56^ were collected. The patient retained enough tactile ability after spinal cord injury to sense short stroking stimuli delivered to his arm and finger. Touch conditions occurred on either the patient’s arm, finger, or an inanimate object, in a variety of multisensory contexts (Table 1). Our results provide evidence that tactile information in S1 is encoded as part of the well-established cortical homunculus, as well as in a more general manner that encompasses larger areas of the body. In addition, we find that S1 does not respond to observed touches to oneself, another person, or an object, but that vision does modulate neural activity when it is paired with physical tactile stimulation. This finding suggests that passively observing visual information depicting touches fails to meet some threshold of relevance or attention necessary to activate S1 neurons.

## RESULTS

**Figure 1.**
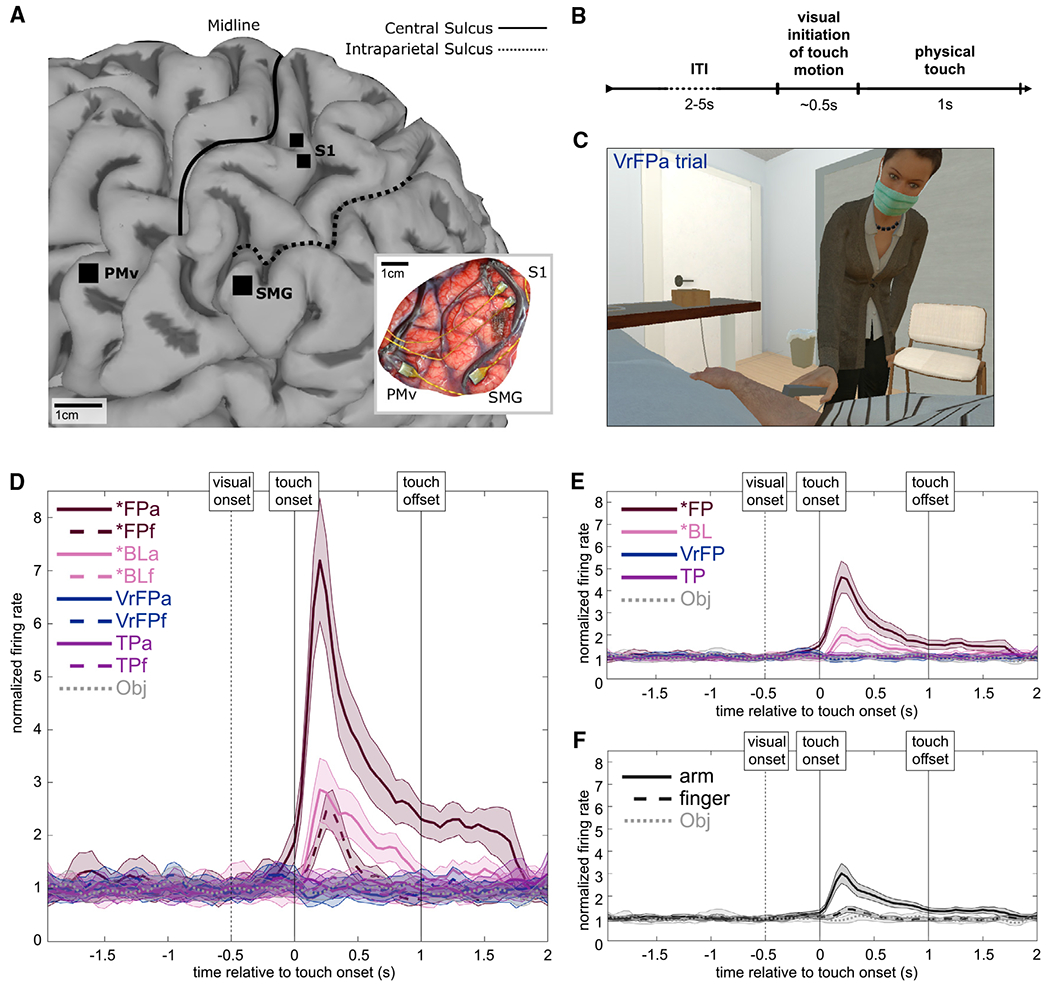
Experimental methods and paradigm Data were recorded in a human tetraplegic participant (n = 1) implanted with microelectrode arrays. (A) Array implant locations on the cortical surface of the left hemisphere, rendered with MRI. Only data from the two S1 arrays were analyzed in this study. Inset: *in situ* array locations. Scale bar: 1 cm. Figure reproduced from Armenta Salas et al.^56^ (B) Task time course. Visual initiation of touch motion was perceived by the participant only in touch types with visual content (*FP, VrFP, TP, Obj). (C) Sample frame from a VrFPa trial, presented using a virtual reality headset. (D) Example smoothed firing rate of one S1 channel to each tested modality of touch (n = 70 trials/modality). Shaded area surrounding each line indicates standard error of the mean (SEM); n = 70 trials per modality. (E and F) Activity of the same channel averaged across modalities to isolate touch type (n = 140 trials except for Obj, where n = 70 trials) and effector (n = 280 trials except for Obj, where n = 70 trials), respectively (i.e., *FPa and *FPf in D are averaged to yield *FP in E). See also Video S1.

S1 responses to visual and tactile stroking stimuli along the arm and finger in a human tetraplegic participant were recorded via two intracortical microelectrode arrays. Within arm and finger locations, neural responses to four touch types were examined (Table 1; Figure 1). A fifth touch type (Obj) used an inanimate object as a control rather than a body location, resulting in a total of nine conditions across locations and touch types. Seventy trials were collected in each condition.

Multi-unit channel activity (Figure 1A) recorded during these trials was aligned to the physical or virtual moment of contact between the touch sensor and the item being touched (touch onset). In visual conditions (first person real physical and visual touch [*FP], first person virtual visual touch [VrFP], third person touch [TP], and object touch [Obj]), visual information predicting the touch was available beginning approximately 0.5 s before touch onset, because the experimenter could be seen beginning the motion toward the touch target (Figure 1B; Video S1). In totality, the task comprised nine conditions (Table 1); the average firing rate of a single channel to each condition is plotted as an example (Figure 1D). The task was designed such that data could be averaged across location (Figure 1E) or averaged across touch type (Figure 1F) to better isolate neural responses to these factors.

### Condition identity decoding

**Figure 2.**
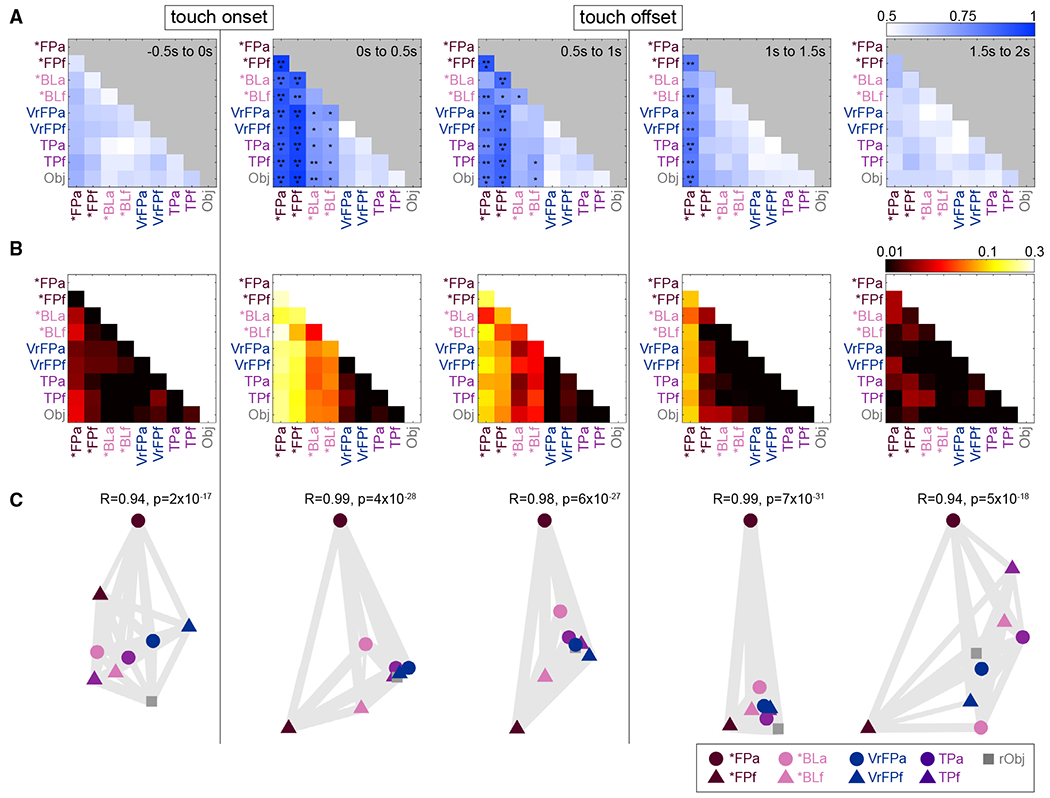
Pairwise decoding and representational similarity analysis (RSA) (A) Pairwise identity decoding results. At each 0.5 s time bin, an LDA classifier was trained to distinguish between each pair of modalities based on the top 40 principal components of multiunit activity. The 70 trials per modality were randomly divided in half to generate train and test data 1,000 times, and the accuracies of the resulting decoders were averaged together to yield the values in the confusion matrices. Asterisks represent significantly different accuracies relative to a null distribution, which was generated by training the same decoder on data with shuffled labels 1,000 times. *Significantly different 95% confidence intervals (CIs); **97.5% CIs; ***99% CIs. (B) RSA was performed on touch-onset-aligned multi-unit S1 channel activity, and resulting representational dissimilarity matrices (RDMs) are shown. Distances between conditions (plotted on log axis) are cross-validated Mahalanobis distance with multivariate noise correction; a distance of zero indicates conditions are statistically indistinguishable. (C) Multi-dimensional scaling (MDS) plots of RDMs in (B). Axes are arbitrary but have been rotated for consistency across time bins. Gray lines between condition icons are “rubber bands” whose thickness is based on the goodness of fit of the scaling. A relatively thinner, more “stretched” band between conditions indicates that in a plot that fully captures neural geometry, the conditions would be closer together. See also Video S2.

Linear discriminant analysis (LDA) was performed on the top 40 dimensions of the multi-unit channel data, sub-selected over 1,000 train/test divisions for equal class sizes and averaged together (Figure 2A). Classifiers were trained on every pair of conditions, using average firing rates binned in 0.5 s increments. No significant decoding occurs prior to touch onset. In the first 0.5 s following touch onset, conditions containing a physical touch (*FP, *BL) can be meaningfully distinguished from purely visual conditions (VrFP, TP, Obj) in all cases and can be significantly distinguished from one another in every case except *BLa vs. *BLf (accuracy = 70% [60%–80%]), which becomes significant only 0.5 s later (72% [61%–81%]). *FPa vs. *BLa is highly decodable with an accuracy of 87.7% (80%–94.3%) despite the two classes varying only on the basis of visual information; similarly, *FPf vs. *BLf obtains an accuracy of 83.7% (74.3%–91.4%).

Overall, in the first time bin after touch onset, *FPa and *FPf are highly distinguishable from other conditions, especially those without physical touch. *BLa and *BLf are less significantly distinguishable. In the following time bin (0.5–1 s), this relative disparity in classification accuracies remains true, but classifications are overall weaker (pairwise one-sided t test across all accuracies, p = 3 × 10^−9^).

In the (1–1.5 s) bin, which occurs immediately after touch offset, *FPa is the only condition that can be distinguished from the other conditions. 1.5 s after touch onset, no classifiers obtain significant decoding accuracy.

To examine decoding on a finer time scale, the same LDA classifiers as described above were run in touch-onset-aligned 0.1 s bins (Video S2). No significant decoding occurs before the 0–0.1 s bin. In this bin, *FPa can be significantly decoded from all conditions apart from *FPf and *BLa, but no other classifiers are significant. In the following 100 ms (0.1–0.2 s after touch onset), *FP/*BL can be significantly distinguished from all other conditions with the exception of *FPf vs. *BLf. By 0.3–0.4 s after touch onset, decoding is overall weaker than in the first bin, suggesting the time period of 0–0.2 s following touch onset contains the strongest touch representations. By 0.7–0.8 s after touch onset, nearly all classifiers cease to be significantly accurate.

### Representational similarity analysis (RSA)

To better visualize the relationships between different task conditions, RSA^57^ was used on the same multi-unit activity as analyzed with the linear classifier (Figure 2B). Representational dissimilarity matrices (RDMs) were computed based on the cross-validated Mahalanobis distance with multivariate noise correction.^58^ For visualization purposes, multi-dimensional scaling (MDS) was used to scale the relationships captured in the RDMs into two dimensions (Figure 2C).^59^

There is a high level of similarity between pairwise decoding (Figure 2A) and the RDMs (Figure 2B) during touch encoding (for the three consecutive time bins after touch onset, r > 0.89; p < 1 × 10^−12^ in all cases, Bonferroni corrected). This similarity is expected because both methods assess the discriminability of neural activity averaged across conditions.

In the 0.5 s prior to touch onset, distances between conditions form a pattern that shares a mild correlation with activity after touch onset; activity is most correlated between the −0.5 to 0 s RDM and the 1–1.5 s RDM (Figure 2B, Pearson correlations between −0.5 and 0 s RDM and RDMs 0–2s, in chronological order: r = 0.67, 0.61, 0.70, and 0.38; p = 3 × 10^−11^, 3 × 10^−9^, 1 × 10^−12^, and 5 × 10^−4^, Bonferroni corrected).

Once touch occurs, the initial RDM within 0–0.5 s after touch onset contains a strong pattern that remains stable during the touch and afterward, although it becomes weaker as time elapses (Pearson correlations between 0 and 0.5 s RDM and RDMs 0.5–2 s, in chronological order: r = 0.96, 0.86, and 0.62; p = 2 × 10^−43^, 1 × 10^−24^, and 9 × 10^−10^, Bonferroni corrected).

Within the 0–0.5 s bin, touch types with only visual stimuli (VrFP, TP, Obj) are less distinguishable and tightly grouped together, whereas the physical touch types (*FP, *BL) are more distinct from one another and therefore more spread out (two-sample t test on distances within VrFP/TP/Obj vs. distances within *FP/*vBL: p = 9 × 10^−5^).

*FP and *BL vary in their level of separation from the non-physical touch types (Figure 2C, 0–0.5 s bin). *FP (mean = 0.17; SD = 0.03) is more distant to the non-physical touch types than *BL (mean = 0.04; SD = 0.03). These sets of distances are significantly different from each other (paired t test, p = 3 × 10^−5^). In addition, during and immediately after the touch, *FP/*BL arm representations are grouped distinctly from the finger representations: on the MDS plots (Figure 2C), arm conditions are consistently grouped separately (above) from the finger conditions.

### Location and touch-type generalization decoding

**Figure 3.**
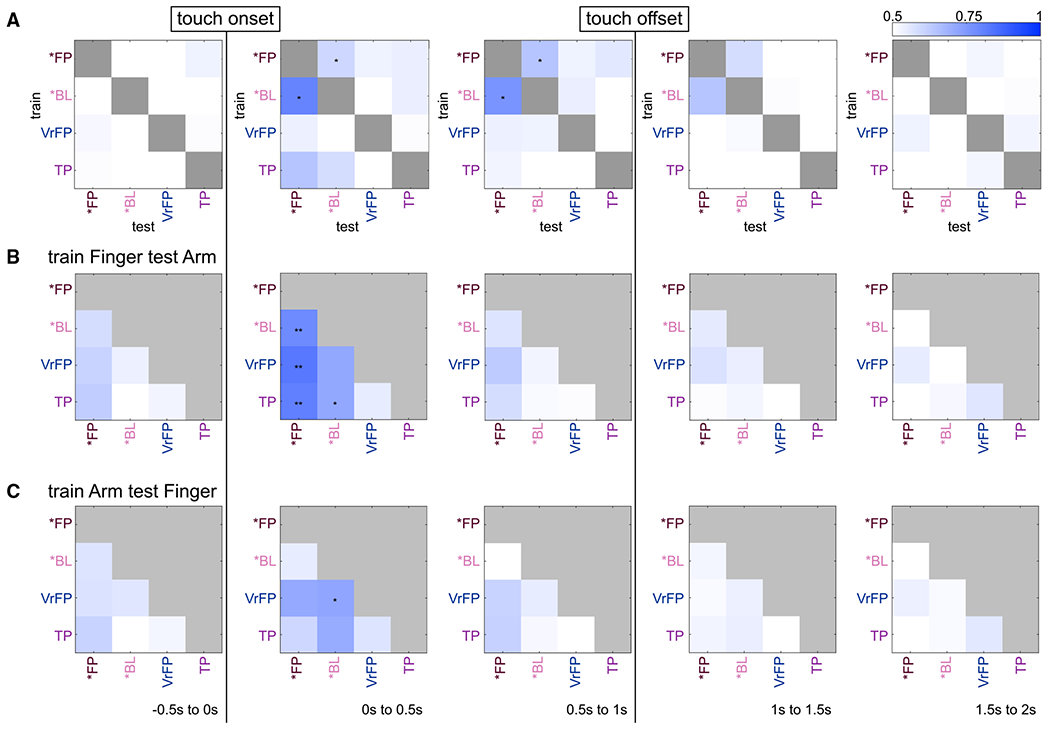
Generalization decoding results (A) Effector was decoded across all pairs of touch types. For example, the bottom left square of each grid represents the average accuracy when a decoder is trained to distinguish arm vs. finger on TP trials and tested on *FP trials. See also Video S3. (B) Pairs of touch types were decoded, training on finger trials and testing on arm trials. For example, the bottom left square of each grid represents the average accuracy when a decoder is trained to distinguish TP vs. *FP on finger trials and tested on arm trials. See also Video S4. (C) The same procedure as in (B) except that training occurred on arm trials and testing on finger trials. See also Video S5. All decoders used 140 trials in training and testing, respectively (70 of each effector). All statistics and plotting conventions are as in Figure 2A.

To investigate whether body location information generalizes across touch types, LDA classifiers were trained to differentiate arm/finger conditions within one touch type and tested on another (Figure 3A). During the touch (0–1 s), body location information generalizes within physical touch conditions; it is possible to train the classifier on *FP and decode body location from *BL, or vice versa. The strongest decoding is achieved in the 0–0.5 s bin by the decoder that trained on *BL and tested on *FP (accuracy = 79.6% [68.6%−90%]). After touch offset, generalization is no longer possible.

When the same decoding problem is performed in 0.1 s bins (Video S3), the only significant accuracies are at 0.1–0.2 s after touch onset for the same subset of conditions significant in the 0–0.5 s bin, indicating a small window of time when body location can generalize strongly across touch types. The opposite question was also interrogated: can a classifier trained on one body location successfully decode the type of touch presented using another body location? In this case, for classifiers that trained on finger data and tested on arm data (Figure 3B) and classifiers that trained on arm data and tested on finger data (Figure 3C), the only significantly decodable instances occur in the 0–0.5 s time bin.

Decoding is notably asymmetric between training on finger/testing on arm and the opposite paradigm: *FP can be strongly distinguished from all other conditions when training on finger and testing on arm but cannot be significantly distinguished from any conditions when training on arm and testing on finger. In addition, there are smaller asymmetries in significance across the two paradigms when distinguishing *BL from VrFP or TP. Decoding in 0.1 s time bins is overall weaker and is significant only in the 0.2–0.3 s time bin for training on finger and testing on arm (Video S4), whereas training on arm and testing on finger never reaches significance at any time point (Video S5).

### Individual channel-tuning analysis

**Figure 4.**
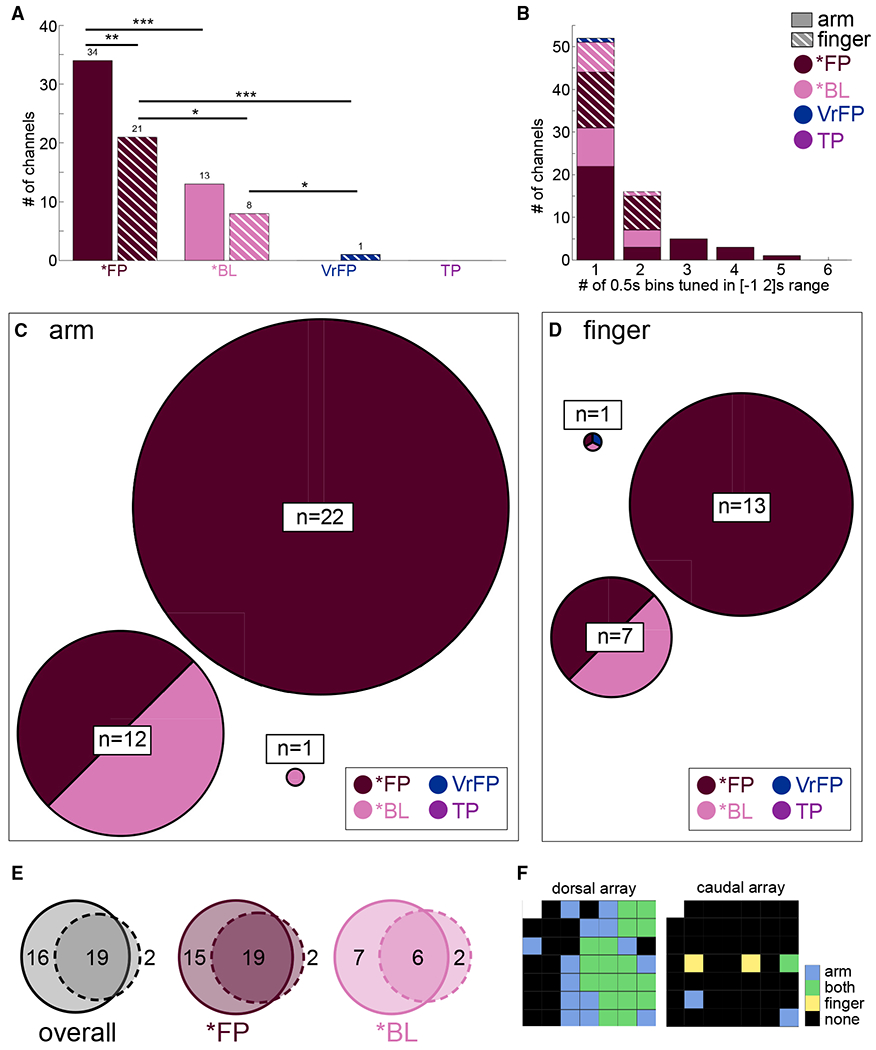
Tuning analysis Channels selective for any touch modality (p < 0.05, Bonferroni-corrected linear regression analysis) at any time bin in the −1 to 2 s range relative to touch onset were examined. (A) Total number of channels tuned within arm and finger touch conditions. Asterisks indicate non-overlapping 95% CIs generated by bootstrapping trials to calculate the distribution of tuned channels. *Significantly different 95% CIs; **97.5% CIs; ***99% CIs. (B) Histogram indicating the range of time that channels were tuned. Tuning was performed in 0.5 s non-overlapping time bins (maximum bins a channel could be tuned to in the −1 to 2 s range was 6). (C) Circles indicate the specific set of modalities each channel was tuned to, within arm touch modalities, and have a diameter proportional to the number n channels tuned to that set. (D) Plotting as in (C) but based on finger touch conditions. (E) Distribution of channels tuned to arm (solid line circle), finger (dashed line circle), or both, across all touch conditions (left) or within touch types (middle and right). (F) Array map of implanted electrodes, indicating locational tuning across all conditions.

To investigate the tuning properties of individual channels within the S1 arrays, linear regression analysis was performed in 0.5 s bins aligned to touch onset. The most channels are tuned to *FPa (34, 95% confidence interval [CI] = [30, 45] of 96 channels total), a number significantly greater than the number of channels tuned to *FPf (21 [18, 26]) or *BLa (13 [10, 22]; Figure 4A). *FPf elicits more tuned channels than *BLf, which trails at 8 [6, 16] channels. Of the non-physical touch conditions, only 1 [1, 8] channel is tuned to VrFPf. The number of time bins that tuned channels are responsive to a given condition is quantified (Figure 4B). No tuning to finger conditions occurs for longer than two bins (1 s). *BLa tuning follows the same rule, but *FPa trials elicit up to five bins (2.5 s) of responsive activity.

The overlap across tuned arm conditions within channels was calculated (Figure 4C). Twenty-two channels are tuned to *FPa solely, whereas 12 channels are tuned to *FPa and *BLa together. Similarly, 13 channels are tuned to *FPf solely, whereas 7 channels are tuned to *FPf and *BLf together. In finger conditions (Figure 4D), fewer channels are tuned overall. Within arm and finger, channels are nearly all tuned to *FP conditions (Figures 4C and 4D).

The overlap of tuned channels across all arm and finger conditions was determined (Figure 4E). Overall, most channels are tuned to both arm and finger (n = 19), whereas 16 channels are tuned only to arm conditions. Only two channels are tuned to solely finger conditions. Within touch types, a similar pattern emerges. In *FP, 19 channels are tuned to both arm and finger, 15 to just arm, and 2 channels to just finger. In *BL, 6 channels are tuned to arm and finger, 7 to just arm, and 2 to just finger. Lastly, the position of tuned channels across all arm and finger conditions was plotted on diagrams of the microelectrode arrays (Figure 4F). Channels tuned to both arm and finger are clustered together, surrounded by channels tuned to arm only. The two exclusively finger-tuned channels are located on the opposite array from the other channels.

**Figure 5.**
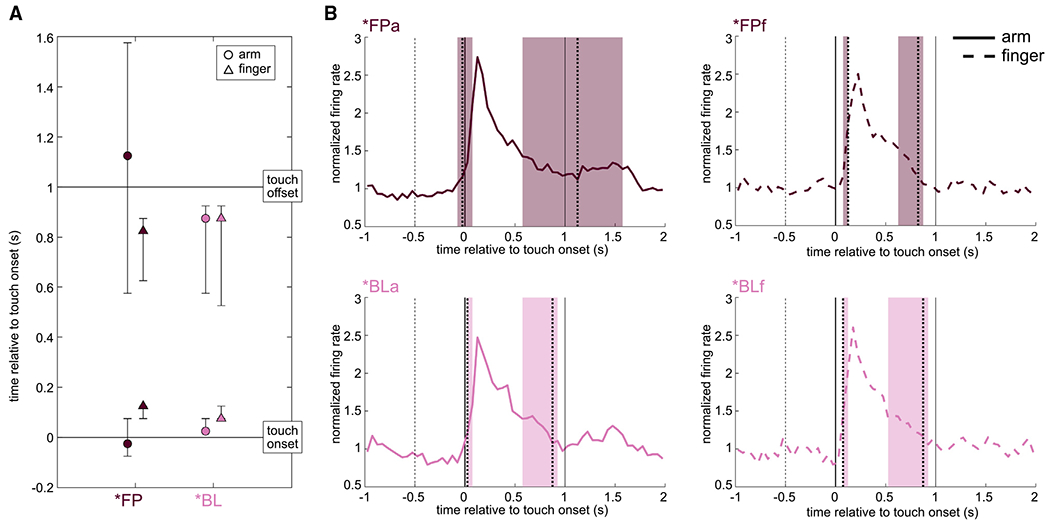
Neural dynamics of tuned channels (A) Onset and offset of channel tuning. Onset of tuning was calculated based on the average normalized firing rate across all tuned channels and condition trials, as the first time bin where average activity exceeded the 95^th^ percentile of the distribution of average baseline responses. Offset was calculated on the same data as the first time bin to dip below the 95^th^ percentile of baseline activity after the peak firing rate. Error bars represent 95% CI obtained by bootstrapping 10,000 times over trials (*FPa: n = 34 tuned channels; *FPf: n = 21; *BLa: n = 13; *BLf: n = 8). (B) Average responses to individual conditions in the same tuned channels as (A), relative to touch onset (vertical black line at 0 s); touch offset is plotted as a vertical black line at 1 s. Vertical dotted lines indicate onset and offset of response with colored background depicting 95% CIs.

The average tuned response curves of channels tuned to *FP and *BL conditions were examined (Figure 5), calculated as deviation from the distribution of baseline activity: *FPa onset = −25 ms (95% CI = −75, 75); *FPf onset = 125 ms (75, 125); *BLa onset = 25 ms (25, 75); and *BLf onset = 75 ms (75, 125). The average offset times of tuned activity were also determined, relative to onset of the touch stimulus: *FPa offset = 1,125 ms (575, 1,575); *FPf offset = 825 ms (625, 875); *BLa offset = 875 ms (575, 925); and *BLf offset = 875 ms (525, 925).

Although there is substantial variance across trials and electrodes, a major trend emerges from this analysis. Within *FP and *BL, arm onset times always occur before finger onset times, whereas offset times are similar across conditions with the exception of the wide variance of *FPa (Figure 5A). Within arm and within finger, onset times are not statistically different, although the mean of *FPa onsets occurs slightly prior to touch onset. In all conditions (Figure 5B), activity peaks sharply immediately following touch onset, followed by a gradual decrement of activity back to baseline.

## DISCUSSION

To examine how S1 represents tactile events based on their location and their multisensory context, electrophysiology data from the putative area 1 of the S1 arm region were examined (Figure 1A). Within arm and finger locations, touch types varying in their tactile and visual content were tested (Table 1). It is worth noting that the participant’s long-term tactile impairment could have resulted in representations of touch in S1 that are altered relative to healthy humans. This is unlikely to be a major effect on the findings of this study, because recent work has shown that topographic representations in S1 are highly preserved in tetraplegic people, even years post-injury, although these representations can weaken over time.^60,61^

Analysis of this rich, exploratory dataset suggested two main conclusions about local neural activity where the multi-electrode arrays were implanted: (1) this S1 area is specialized for arm representations but is capable of representing touch information from the finger in a more general manner, and (2) this S1 area is modulated by vision during physical touches but is not activated by vision on its own.

### Neural activity is specialized for arm touches and represents finger touches more generally

Immediately after touch onset in FP* and BL*, arm conditions are separated from finger conditions, based on neural activity visualized using MDS (Figure 2C). This division based on touch location continues until the touch ceases. *FP and *BL are both separable by touch location, although *BL is significantly less separable. This pattern is also evident in the linear decoding analysis, where *FPa vs. *FPf is immediately highly decodable on touch onset, whereas *BLa vs. *BLf is significantly decodable only one time step (0.5 s) later (Figure 2A). *FPa can be distinguished from all other conditions for much longer than any other condition, up to 1.5 s after touch onset, and is the first condition to become decodable in the 0.1 s bin classifier (Video S2).

Despite *FP seeming to contain more robust location information than *BL, classifiers trained on either *FP or *BL and tested on the other to distinguish arm from finger trials achieve significant decoding (Figure 3A). Locational information is therefore sufficiently present in *BL conditions to allow for generalization to and from *FP conditions. Through analysis of the 0.1 s bin decoders, it appears the bulk of this locational information is present 0.1–0.2 s after touch onset.

Although arm and finger touches are both represented in S1, an asymmetry becomes apparent when training classifiers to decode touch type while generalizing across body locations. In particular, *FP trials can be distinguished from all other touch types when classifiers are trained on finger data and tested on arm data (Figure 3B), but the reverse is not true (Figure 3C): *FP trials are indistinguishable from other touch types when classifiers are trained on arm data and tested on finger data. Because the data were recorded in a putative arm area, it is likely that this asymmetry is due to different levels of encoding specificity. Arm touches may be represented in a highly specific manner that does not generalize to other touch locations, whereas finger touches (and potentially touches from other areas) may be represented more generally as they are outside of their primary topographic S1 location.

The tuning analysis further demonstrates the differences in arm and finger neural representations. More channels overall are tuned to arm than finger conditions (Figure 4A), and *FPa trials elicit tuning forupto five time bins (2.5s), whereas *FPf tuninglastedonlyup to two time bins (1 s; Figure 4B). The vast majority of tuned channels are either tuned to solely arm conditions or both arm and finger conditions (Figure 4E). Only two channels are tuned to solely finger conditions, suggesting the bulk of the neural population recorded is not selective to finger touches specifically but may be activated by body touches more generally in addition to arm touches specifically. *FP and *BL each contain roughly equivalent numbers of channels tuned to solely arm or to both finger and arm, and very few channels tuned solely to finger (Figure 4E). Onset analysis of *FP and *BL reveal a trend that appears to mesh with this pattern: *FPa onset occurs 0.15 s before *FPf onset, and *BLa onset occurs 0.05 s before *BLf (Figure 5A). In other words, arm conditions elicit sharply tuned neural responses that begin before the tuned responses to finger conditions (Figure 5B).

To summarize, neural activity elicited by physical touches delivered to the arm forms patterns distinct from the activity elicited by touches to the finger. Individual channels tend to be tuned to both arm and finger, or just arm conditions, but rarely just finger conditions. Tuned activity starts earlier for arm conditions than finger conditions. This evidence builds a picture of a region of S1 that is primarily geared toward representing arm touches. A neural sub-population of this region is also capable of representing finger touches, albeit less strongly or specifically.

There could be several reasons for this difference between the two tested locations. One is that, due to the spinal cord injury, the participant was able to sense one location more strongly and naturalistically than the other. The patient reported finger sensations to be more natural, yet neural finger representations were weaker. This makes the participant’s uneven tactile impairment an unlikely culprit for the differences in location encoding.

If the differences between arm and finger representations are not primarily due to differences in spinal cord damage and tactile impairment, then they are likely due to differences in the neural representations of these locations. The distribution of tuned channels appears geographically distributed when mapped to the implanted micro-electrode arrays: the upper array contains the bulk of activity, with a nucleus of channels tuned to both arm and finger conditions and a surrounding of channels tuned solely to arm conditions (Figure 4F). The only two finger-specific channels are located on the lower array. Cortical curvature may have resulted in electrodes recording from varying cortical layers within S1.

From prior work with this participant, it is known that intracortical microstimulation (ICMS) of the S1 arrays studied here elicits cutaneous and proprioceptive sensations primarily in the arm, with a much smaller number of sensations in the fingers.^56^ It is likely the arrays, especially the dorsal array (Figure 4F), are located in the arm region of area 1 within S1. The neural response to finger touches detailed here contribute to the growing literature suggesting that although S1 overall does maintain a gross representation of the body along the lines of the homunculus laid out in the earliest human cortical stimulation studies and observed many times since,^17–21^ it also contains other more complex levels of tactile representations.^50–54^ Most recently, Muret et al.^54^ used magnetic resonance imaging (MRI) to show that different body locations are represented in S1 in areas beyond their primary topographic area both in area 3b specifically and S1 overall. Our findings support and expand this finding, indicating that S1 area 1 encodes highly specific and rapid responses to touches through its established topography, but tactile information from other anatomical areas outside this topography may activate area 1 in a more general manner.

### Visual information modulates neural activity if accompanied by a physical stimulus

S1 neural activity is restricted to conditions that contain a physical tactile stimulus, and less than 2% of channels are tuned to visual-only conditions (Figure 4A). Although several variations on visual touches without physical stimuli were tested (VrFP, TP, Obj), they are not represented in a discriminable manner from one another in S1 (Figure 2). VrFP and TP do not elicit representations of touch location information in S1 activity, whether decoded in an identity or generalization problem (Figures 2A and 3A). Across all methods in this study, there is no detectable encoding of tactile information in S1 from purely visual stimuli.

In contrast, *FP trials contain visual information paired with a physical stimulus and, immediately after touch onset, they can be easily discriminated from all non-physical conditions and from *BL trials that contain the same physical stimulus minus the visual content (Figure 2A). The strong performance of *FPa vs. *BLa and *FPf vs. *BLf classifiers indicate the presence of visual information is sufficient to change the touch encoding in S1. Visual information also appears to affect the length of time a touch representation occurs in S1, because *FPa is decodable for much longer than any other condition.

RSA demonstrates that the pattern of responses immediately prior to touch onset is mildly correlated with activity during the touch itself, suggesting there is some effect of a visual approach of a tactile stimulus before an expected touch occurs (Figure 2B). However, a much stronger stable pattern of activity emerges once the touch actually begins, as indicated by the correlations between the RDM of the first 0.5 s and the following RDMs. This relationship is evident in the MDS plots generated based on neural activity (Figure 2C). In the second following touch onset, *FP and *BL conditions are separated from all other touch types and from each other. In particular, *FPa and *FPf are highly dissociated from the other conditions. The presence of visual information generalizes across touch location to some extent: a classifier trained on finger trials can distinguish *FP vs. *BL in arm trials, but not vice versa (Figures 3B and 3C). The ability to decode visual information in a manner that generalizes across body location also appears to be present quite late relative to touch onset; the 0.1 s bin decoder achieves any significance only in the 0.2–0.3 s time bin relative to touch onset. These findings speak to a more general distinction between visual and blind physical touches existing in S1 finger touches, which is overridden by more specific information in arm touches that are not able to generalize to other body parts.

There are large populations of channels tuned to *FPa and *FPf, and within these populations there are sub-groups also tuned to *BLa and *BLf, respectively (Figures 4C and 4D). Blind and visual touches appear to activate the same population of neurons, but touches with a visual component activate additional neurons on top of this population.

These results suggest that visual information is enough to distinguish two otherwise identical physical touches in S1, but visual information on its own, whether it relates to oneself (VrFP), another person (TP), or an inanimate object (Obj), is not sufficient to engage S1. This finding is especially intriguing because although it is clear that visual information affects experiences of touch,^11,38,62^ a rapidly evolving scientific literature is still deciding the role of vision in modulating S1.^22,26,27,29–33,63^

The results presented here examine the effect of vision on S1 using human electrophysiology, specifically in a highly localized sub-region of S1, with high spatial resolution of spiking activity. The bulk of prior literature has used functional MRI (fMRI), MEG, and EEG to address this question, data that capture whole-brain dynamics at a relatively low spatial resolution, and likely include membrane potentials that do not produce spikes. These experiments have for the most part examined S1 as a whole, and results have varied, finding either that S1 has no response to observed touch^31–33^ or does respond to observed touch.^22,27,29,30,63^ From the studies examining Brodmann areas more specifically, we see evidence that area 3b^25^ and areas 1 and 2^26^ are capable of responding to observed touch. One potential method to reconcile these findings and the results found here in area 1 would be to examine the type of task employed.

The majority of experiments finding S1 modulation to observed touches employ a touch-relevant task during data collection, whether it be counting touches,^24,29^ answering qualitative questions about the touch type,^23,25,26,30^ or rating touch intensity.^22^ The experiments that find no effect of observed touch on S1 tend to employ either non-touch-related tasks^31^ or simply ask participants to passively observe the stimuli.^32,33^ Thus it is possible that a relevance threshold, modulated by higher-order brain areas, must be exceeded in order for S1 to represent observed touches.^43^ If this is true, the fact that S1 does not respond to visual stimuli when the participant passively observes touches in this study agrees with the existing literature, despite the differences in data types. This effect could also explain why visual information does modulate tactile representations of physical touch: the physical component of the touch activates S1 as it would in a blind touch, but additionally higher-order areas integrate the visual input as sufficiently relevant to the tactile input such that vision affects S1 simultaneously.

What might be the role of this modulation? It is known that vision modulates experiences of touch in a variety of ways, including effects such as visual enhancement of touch^14,35–38^ in which non-informative vision of a body part improves tactile perception. Our results suggest that touch-relevant visual information elicits an earlier tuned response over more neurons and results in a representation of touches that are highly distinguishable in terms of location and multisensory content. All of these attributes have the potential to contribute to visual enhancement of touch. Indeed, these results agree with prior literature that has suggested that S1 is modulated by paired visual and tactile stimuli,^40–43,64,45,63,65,66^ and has also shown that S1 is a necessary component of body-centered visuotactile integration^46–48^ and reflects predictions of tactile events from visual signals.^67^

S1 can be modulated by concepts as high level as affective significance, as was shown in a study that examined the effect of perceived gender of a person delivering a caress to heterosexual men.^62^ S1 is also affected by motor planning (presumably expecting the sensory consequences of upcoming actions)^68,69^ and by imagining touch sensations.^70,71^ It is likely that when S1 is modulated by visual information, it is not directly interacting with the visual system but instead affected by upstream areas that are implementing some version of a forward model to determine expected tactile inputs.

## Conclusions

This study represents a broad exploration of how different types and locations of touch affect a small area in the putative arm region of S1. It contributes to the growing body of literature suggesting that area 1 within S1 contains highly specific topographic organization as classically depicted, but additionally encodes touches outside this topography in a less specific manner. We also find that visual information depicting touches, either to oneself, to another person, or to an object, are not sufficient to activate S1 in a measurable way. However, a blindly sensed physical touch and a visually seen physical touch are represented distinguishably in S1; both elicit strong responses that share commonalities, such as how touch location is encoded, but they are not identical.

Taken as a whole, these findings demonstrate that S1 contains a nuanced and complex encoding of tactile experiences that is to some degree multisensory. Future endeavors should aim to examine these same conditions in a larger population of individuals, both healthy and with a variety of levels of sensorimotor impairment. There are many practical applications for a better understanding of S1, including the improvement of restorative devices seeking to artificially generate tactile sensations in deafferented limbs and prosthetics.^56,72–74^ By better understanding how naturalistic tactile sensations are encoded in S1, and how they interact with cues from other sensory modalities, we can improve our ability to generate biomimetic artificial stimuli.

### Limitations of the study

The dataset examined here, although informative, is limited in several ways. Recordings from more locations of S1 would have allowed for a better understanding of the differences between Brodmann areas 1, 2, 3a, and 3b within S1, as well as differences along the topographic map within areas. Examining only two body parts leaves room for the possibility that other body parts are represented differently than the ones tested; because array localization was based on subjective ICMS responses,^56^ these locations have some uncertainty and limited precision. Visual information within the task may have contributed to a variety of processes, including expectation/prediction of touch onset, face processing, peri-personal space processing, and attentional factors. The different conditions tested here may be more or less salient, but these differences occur as part of our biologically relevant task design, and part of the experiment was explicitly addressing how different visuotactile contexts affect S1. Data from only one participant can confound individual differences with population trends, and although unlikely, it is possible the participant’s spinal cord injury has caused some remapping of S1.^60,61^ Finally, due to restrictions on data collection, recordings were collected over the course of 6 months, and some conditions were tested in separate sessions, which may have affected decoding and introduced confounds associated with neural recordings drifting overtime.

## STAR★METHODS

### RESOURCE AVAILABILITY

#### Lead contact

Further information and requests for resources and reagents should be directed to and will be fulfilled by the lead contact, Isabelle A. Rosenthal (rosenthalia@caltech.edu).

#### Materials availability

This study did not generate any new unique reagents.

#### Data and code availability

- Original data is available at Zenodo and is publically available as of the date of publication. The DOI is listed in the key resources table.
- All original code has been deposited at Zenodo and is publically available as of the date of publication. The DOI is listed in the key resources table.
- Any additional information required to reanalyze the data reported in this paper is available from the lead contact upon request.

### EXPERIMENTAL MODEL AND SUBJECT DETAILS

A C5-level incomplete tetraplegic participant (male, 32 years old) was recruited and consented for a brain-machine interface (BMI) clinical trial including intracortical recording and stimulation. At the beginning of data collection, the participant was 6.5 years post-injury and 5 years post-implant. All procedures were approved by the Institutional Review Boards (IRB) of the California Institute of Technology, University of Southern California, and Rancho Los Amigos National Rehabilitation Hospital.

### METHOD DETAILS

#### Implants

The participant was implanted with microelectrode arrays in three locations in the left hemisphere: the supra-marginal gyrus (SMG), ventral premotor cortex (PMv), and primary somatosensory cortex (S1) (Figure 1A). This paper only examines data in S1, which was recorded using two 48-channel 1.5mm SIROF-tipped (sputtered iridium oxide film) microelectrode arrays (Blackrock Microsystems, Salt Lake City, UT). Given the curvature of sensorimotor cortex and the need to implant arrays on the gyral surface, it is likely the S1 micro-electrode arrays are located in Brodmann area 1 (BA 1). Additional details pertaining to the arrays and the specifics of surgical planning are described in.^56^

#### Experimental paradigm

Two anatomical locations were examined across a set of tactile and visual conditions. The two locations selected were a “finger” location on the back of the thumb where the participant reported naturalistic sensations, and an “arm” location near the back of the elbow where the participant reported numb sensation. These locations were selected on the basis of a preliminary mapping of the participant’s tactile capabilities on the arm and hand using Semmes-Weinstein filaments at varying strengths, which took place two days prior to the first experimental session.

Although the different task conditions (Table 1) did not all include both a physical and a visual component, all employed the same style of touch: a 1-second stroke over approximately 6cm of skin. The touch was delivered by a plastic rod (or a virtual facsimile of one), built in-house, which had a raised button on one end (1.5 x 2 cm) that was passed along the touch location. The rod housed a load cell which was used to record the pressure applied and align the onset of touch to neural recordings.

Each trial consisted of an inter-trial-interval (ITI) of 5s with an additional 0-3s jitter, followed by a 1s touch stimulus and 1s post-touch phase. In trials with a visual component (approach towards the touch target), this component began approximately 0.5s before touch onset. The experimenter performing the touch was positioned at approximately a one o’clock position relative to the participant’s head, such that the participant could clearly see the experimenter and the approach trajectory of the touch stimulus (Figure 1C and Video S1). The uniform and direct nature of the approach trajectory meant that the participant could approximately anticipate when a touch would begin using visual information once the approach began. A total of 11 conditions were examined, incorporating 6 touch types and 3 touch locations (Table 1).

#### Data collection

Neural data was recorded from each microelectrode array using a 128-channel Neural Signal Processor (Blackrock Microsystems) as 30,000 Hz broadband signals. Data was collected in 8 sessions over 6 months, in two sets (see Table 1 for task condition descriptions). In the first set, the participant observed real physical touches to his body in first person (*FP), the same touches delivered to someone else (third person; TP), and touches to an inanimate object (Obj). This set was collected over the first two months in 4 sessions with up to 3 weeks between sessions. In the second set, the participant experienced real physical touches without visual touch information (blind; *BL), and saw touches being delivered to him in first person using virtual reality, without any physical touch component (VrFP). The second set was collected over the third to sixth months in 4 sessions with up to 9 weeks between sessions.

Within a session, data was collected in series of 11-trial runs. Each run contained 10 trials of the same condition and one catch trial. Within the two sets, runs were pseudorandomly shuffled so there were no two runs of the same condition back to back in any session. 1-2 runs of each condition within a set were collected in each session. 70 trials (7 runs) were collected in every condition. At the start of each run, the participant was informed which type of stimuli would be delivered and was instructed to attend to the stimuli while visually fixating on the touch location except for BL trials in which he fixated on a non-informative dot centered in his field of view.

In third person (TP) trials, the third person being touched (an experimenter familiar to the participant) was positioned so their arm and hand were adjacent and parallel to the participant’s own arm and hand. In object (Obj) trials, the participant observed a wooden block approximately the size of his hand being touched along its flat surface while it lay on a desk in front of him.

The conditions in set two required a virtual reality headset; a Vive Pro Eye was used to display a virtual environment run with Unity, which closely mimicked the data collection room and gave the participant a first-person perspective over a virtual body with a size, gender, and posture reflecting his own body. In the virtual environment, a virtual experimenter was animated to deliver touches in a manner resembling the real experimenter (Figure 1C, Video S1). The human avatar for the virtual experimenter was taken from the Microsoft Rocketbox Avatar Library^75^ (https://github.com/microsoft/Microsoft-Rocketbox/). For *BL conditions, the headset was used as a blindfold, and displayed a non-informative white dot in the center of a black field of view which the participant was instructed to fixate on.

To verify that the fundamentals of the neural signal remained unchanged across the two sets, SNR was analyzed using two different metrics. 1) In every run, the ratio of the mean waveform’s peak value on each channel to the root mean square of the noise estimate for that channel was computed. 2) The ratio of the mean waveform on each channel to the standard deviation of the waveform within every run was also computed. Both these metrics were averaged across runs within each set; neither metric was different across the two sets (Wilcoxon sign rank test, 1) p=0.28; 2) p=0.08).

The mean and standard deviation of ITI firing rates taken from the time period [4s to 1s] before each touch stimulus began, were also examined. All firing rates were normalized by dividing by the mean of the baseline within a run, then averaged across all trials and runs within a set and compared across sets. Both the mean (Wilcoxon sign rank test, p=0.18) and the standard deviation (p=0.53) of ITI firing rates were not significantly different between sets.

Although outside the scope of this paper, additional conditions were collected along with the ones analyzed here. In set one, conditions in which the participant imagined the touches being delivered without any external tactile stimuli were also obtained. In set two, a touch type identical to VrFP except that the participant’s virtual body was composed of abstract blocks rather than a realistic human body was collected, and a condition in which the participant viewed an inanimate object being touched in virtual reality was also acquired.

### QUANTIFICATION AND STATISTICAL ANALYSIS

All analyses were performed using MATLAB R2019b (MathWorks, Natick, MA) unless otherwise indicated.

#### Preprocessing and temporal alignment of data

Firing rates for each electrode were extracted in 50ms bins from the broadband signal in a multi-unit, unsorted fashion,^76,77^ using a threshold of −3.5 times the noise RMS of the continuous signal voltage. This multi-unit channel activity was aligned within each trial to the physical or virtual moment of contact between the touch sensor and the item being touched (i.e. touch onset). In conditions with a physical touch component, touch onset was calculated using the pressure readings obtained from the rod used to deliver touches; in conditions with only a virtual touch component, touch onset was calculated using the timing of Unity animations.

To normalize firing rates, within each run and each channel, a mean baseline firing rate was calculated from the time period 4s to 2.5s prior to each touch onset and averaged across trials. The firing rates of each channel at every time point were divided by this baseline.

#### Decoding analysis

Linear Discriminant Analysis (LDA) pairwise classifiers were used to probe the linearly decodable information within and across task conditions (Figures 2A and 3). Normalized firing rate data was binned into either 0.5s or 0.1s bins, depending on the analysis. Within each bin, data was randomly split equally into train/test partitions, regardless of session collected. This split occurred 1000 times and was balanced each time to include equal numbers of trials from every condition tested (70 trials per condition = 35 trials each in train and test).

Singular value decomposition (SVD) was used to perform dimensionality reduction on the initial 96 multi-unit channels of the training dataset. Average firing rate data from both train and test datasets in each bin was projected on the top 40 features capturing the most variance in the training data.

LDA classifiers were fit to the resulting data using MATLAB’s *fitcdiscr* function across the 1000 iterations. The overall performance of each classifier was taken as the average performance and 95% confidence intervals on this estimate were taken from the distribution of accuracies across iterations. This analysis was repeated on a null dataset in which condition labels were shuffled across trials in order to generate chance-level performance of the classifier. Significance was calculated by comparing the accuracy percentile values of the classifiers with their null counterparts.

#### RSA and MDS

Representational Similarity Analysis (RSA) was employed on normalized firing rate data to assess the relationships between touch conditions (Figures 2B and 2C).^57,59^ Cross-validated Mahalanobis distance with multivariate noise normalization was used as the measure of dissimilarity.^58^ The noise covariance matrix was estimated from the data and regularized toward a diagonal matrix to ensure that it would be invertible. The cross-validated Mahalanobis distance is an unbiased measure of square Mahalanobis distance with the added benefit of having a meaningful zero-point.^58,78^ The larger the Mahalanobis distance between two conditions, the more discriminable their neural patterns. If the patterns are fully indiscriminable, their distance is 0. This continuous measure is directly related to discrete classification performance with pairwise LDA. Cross-validated Mahalanobis distance is thus less affected by common activation patterns across conditions in comparison to other measures such as Pearson correlation. The python package *rsatoolbox* (https://github.com/rsagroup/rsatoolbox) was used to compute noise covariance and generate representational dissimilarity matrices (RDMs).

Data was cross-validated across 7 splits, divided by the 10-trial runs the data was originally collected in, and RDMs were generated independently on data divided into 0.5s bins. The resulting RDMs were symmetric across the diagonal, with meaningless values on the diagonal itself.

RDMs were visualized with multi-dimensional scaling (MDS) using the MATLAB toolbox *rsatoolbox* (https://github.com/rsagroup/rsatoolbox_matlab).^59^ MDS allows for distances in RDMs to be visualized intuitively in a lower-dimensional space while preserving these distances as much as possible. The MDS visualizations used a metric stress criterion to arrange conditions without assuming any category structure a priori. The stress is visualized on MDS plots (Figure 2C) in the form of gray “rubber bands” stretched between points – the thinner the band, the more the true distances between points are distorted by the low dimensional MDS mapping to be further apart than in the high dimensional RDM.

#### Tuning and onset analysis

Tuning properties of multi-unit channels were assessed via linear regression analysis. In each 500ms bin corresponding to 1s before touch onset to 2s after touch onset, normalized firing rates for each channel were fit to a linear regression model based on the following equation:

$$F=\beta_{0}+\beta_{1}X_{1}+\beta_{2}X_{2}+\ldots \beta_{C}X_{C}$$

where F = vector of firing rates on each trial, X = one-hot-encoded matrix signaling condition identity for each trial, *β* = estimated regression coefficients indicating level of tuning to each condition, and C = number of conditions tested. In addition to data from every trial, F also included 70 entries (to match the number of trials per condition), corresponding to *β*_0_, containing the baseline firing rate of the channel across all trials. This baseline was calculated as a mean of channel activity 4s–2.5s before touch onset in every trial. For each channel and condition fit with linear regression, a student’s t test was performed to assess the null hypothesis *β* = 0. If the null hypothesis was rejected, the channel was determined to be tuned to that condition in comparison to its baseline firing rate. p values were corrected for multiple comparisons using the Bonferroni-Holm method within each channel.

A bootstrap analysis was run for 1000 iterations, in which all conditions were randomly sampled with replacement to yield 70 trials each, to assess significant differences in numbers of tuned channels across conditions (Figure 4).

The channels identified as tuned to any condition in the period of 1s before touch onset to 2s after touch onset were analyzed to determine the average timing onsets and offsets of their tuned responses. Within a condition, firing rates of all tuned channels were averaged together in 50ms bins, and the 95^th^ percentile of the distribution of average baseline firing rates was computed. The onset time for the condition was the middle of the first time bin in which the firing rate rose above the 95^th^ percentile of the average baseline. The offset time was calculated as the middle of the first time bin in which the firing rate dipped below the 95^th^ percentile of the average baseline, after onset. 95% confidence intervals were constructed for the onset and offset times by bootstrapping over trials within the tuned channels 10,000 times.

### ADDITIONAL RESOURCES

The data in this manuscript was collected as part of a clinical trial (NCT01964261).

Clinical trial registry link: https://clinicaltrials.gov/ct2/show/NCT01964261.

## Supplementary Material

1

2

3

4

5

## Figures and Tables

**Table 1.** Experimental task conditions

Modality	Location	Touch type	Description
*FPa	arm	*FP	The participant observed, in first person (FP) perspective, a real physical touch to his arm.
*FPf	finger	*FP	The participant observed, in first person (FP) perspective, a real physical touch to his finger.
*BLa	arm	*BL	The participant fixated on a non-informative dot in virtual reality, effectively blindfolding (BL) him during a real physical touch to his arm.
*BLf	finger	*BL	The participant fixated on a non-informative dot in virtual reality, effectively blindfolding (BL) him during a real physical touch to his finger.
VrFPa	arm	VrFP	The participant observed, via virtual reality and in first person perspective (VrFP), a touch to his arm without any physical contact.
VrFPf	finger	VrFP	The participant observed, via virtual reality and in first person perceptive (VrFP), a touch to his finger without any physical contact.
TPa	arm	TP	The participant observed, in third person (TP) perspective, a real physical touch to another person’s arm.
TPf	finger	TP	The participant observed, in third person (TP) perspective, a real physical touch to another person’s finger.
Obj	object	Obj	The participant observed a real physical touch to an inanimate object (Obj), a wooden block.

Nine different touch modalities were tested. Each modality with a physical touch stimulus is coded with an asterisk (*). All modalities with a stimulus on the arm end in “a”; all modalities with a stimulus on the finger end in “f.” Touches were single strokes delivered by an experimenter using a pressure-sensing rod; in *BL and VrFP trials, the participant wore a Vive Pro Eye headset.

**Table:** KEY RESOURCES TABLE

REAGENT or RESOURCE	SOURCE	IDENTIFIER
Deposited data		
Preprocessed study data	this paper	https://doi.org/10.5281/zenodo.7470655
Software and algorithms		
Analysis code	this paper	https://doi.org/10.5281/zenodo.7470579
MATLAB R2019b	MathWorks	http://www.mathworks.com
Python rsatoolbox	Walther et al.^58^; GitHub	https://github.com/rsagroup/rsatoolbox
MATLAB rsatoolbox	Nili et al.^59^; GitHub	https://github.com/rsagroup/rsatoolbox_matlab
Unity	Unity Technologies	https://unity.com/
Microsoft Rocketbox Avatar Library	Gonzalez-Franco et al.^75^; Microsoft; GitHub	https://github.com/microsoft/Microsoft-Rocketbox/
Other		
Neuroport System	Blackrock Neurotech	https://blackrockneurotech.com/

## Highlights

- We examine human multi-unit electrophysiological data during visuotactile touches
- Vision strongly modulates S1 activity during physical touches
- S1 does not respond to passively viewing touches with no physical stimulus
- Despite classic S1 topography, arm and finger are both represented in S1 arm area

## References

[1] Ghez C, Gordon J, and Ghilardi MF (1995). Impairments of reaching movements in patients without proprioception. II. Effects of visual information on accuracy. J. Neurophysiol 73, 361–372. 10.1152/jn.1995.73.1.361. 7714578

[2] Miall RC, Afanasyeva D, Cole JD, and Mason P (2021). The role of somatosensation in automatic visuo-motor control: a comparison of congenital and acquired sensory loss. Exp. Brain Res 239, 2043–2061. 10.1007/s00221-021-06110-y. 33909112 PMC8282580

[3] Miall RC, Rosenthal O, Ørstavik K, Cole JD, and Sarlegna FR (2019). Loss of haptic feedback impairs control of hand posture: a study in chronically deafferented individuals when grasping and lifting objects. Exp. Brain Res 237, 2167–2184. 10.1007/s00221-019-05583-2. 31209510 PMC6675781

[4] Robles-De-La-Torre G. (2006). The importance of the sense of touch in virtual and real environments. IEEE Multimedia 13, 24–30. 10.1109/MMUL.2006.69.

[5] Sainburg RL, Ghilardi MF, Poizner H, and Ghez C (1995). Control of limb dynamics in normal subjects and patients without proprioception. J. Neurophysiol 73, 820–835. 10.1152/jn.1995.73.2.820. 7760137 PMC11102602

[6] Ehrsson HH (2020). Multisensory processes in body ownership. In Multisensory Perception (Elsevier), pp. 179–200. 10.1016/B978-0-12-812492-5.00008-5.

[7] Jeannerod M. (2003). The mechanism of self-recognition in humans. Behav. Brain Res 142, 1–15. 10.1016/S0166-4328(02)00384-4. 12798261

[8] Tsakiris M, Carpenter L, James D, and Fotopoulou A (2010). Hands only illusion: multisensory integration elicits sense of ownership for body parts but not for non-corporeal objects. Exp. Brain Res 204, 343–352. 10.1007/s00221-009-2039-3. 19820918

[9] Ernst MO, and Banks MS (2002). Humans integrate visual and haptic information in a statistically optimal fashion. Nature 415, 429–433. 10.1038/415429a. 11807554

[10] Kording KP, and Wolpert DM (2004). Bayesian integration in sensorimotor learning. Nature 427, 244–247. 10.1038/nature02169. 14724638

[11] Botvinick M, and Cohen J (1998). Rubber hands ‘feel’ touch that eyes see. Nature 391, 756. 10.1038/35784. 9486643

[12] Johnson RM, Burton PC, and Ro T (2006). Visually induced feelings of touch. Brain Res. 1073–1074, 398–406. 10.1016/j.brainres.2005.12.025. 16455063

[13] Kandula M, Hofman D, and Dijkerman HC (2015). Visuo-tactile interactions are dependent on the predictive value of the visual stimulus. Neuropsychologia 70, 358–366. 10.1016/j.neuropsychologia.2014.12.008. 25498404

[14] Tipper SP, Phillips N, Dancer C, Lloyd D, Howard LA, and McGlone F (2001). Vision influences tactile perception at body sites that cannot be viewed directly. Exp. Brain Res 139, 160–167. 10.1007/s002210100743. 11497057

[15] Ghazanfar AA, and Schroeder CE (2006). Is neocortex essentially multisensory? Trends Cogn. Sci 10, 278–285. 10.1016/j.tics.2006.04.008. 16713325

[16] Delhaye BP, Long KH, and Bensmaia SJ (2018). Neural basis of touch and proprioception in primate cortex. Compr. Physiol 8, 1575–1602. 10.1002/cphy.c170033. 30215864 PMC6330897

[17] Ejaz N, Hamada M, and Diedrichsen J (2015). Hand use predicts the structure of representations in sensorimotor cortex. Nat. Neurosci 18, 1034–1040. 10.1038/nn.4038. 26030847

[18] Kaas A, Goebel R, Valente G, and Sorger B (2019). Topographic somatosensory imagery for real-time fMRI brain-computer interfacing. Front. Hum. Neurosci 13, 427. 10.3389/fnhum.2019.00427. 31920588 PMC6915074

[19] Kolasinski J, Makin TR, Jbabdi S, Clare S, Stagg CJ, and Johansen-Berg H (2016). Investigating the stability of fine-grain digit somatotopy in individual human participants. J. Neurosci 36, 1113–1127. 10.1523/JNEUROSCI.1742-15.2016. 26818501 PMC4728720

[20] Penfield W, and Boldrey E (1937). Somatic motor and sensory representation in the cerebral cortex of man as studied by electrical stimulation. Brain 60, 389–443. 10.1093/brain/60.4.389.

[21] Sanders Z-B, Wesselink DB, Dempsey-Jones H, and Makin TR (2019). Similar somatotopy for active and passive digit representation in primary somatosensory cortex. Neuroscience. 10.1101/754648. PMC10203813 37145934

[22] Blakemore S-J, Bristow D, Bird G, Frith C, and Ward J (2005). Somatosensory activations during the observation of touch and a case of vision-touch synaesthesia. Brain 128, 1571–1583. 10.1093/brain/awh500. 15817510

[23] Bufalari I, Aprile T, Avenanti A, Di Russo F, and Aglioti SM (2007). Empathy for pain and touch in the human somatosensory cortex. Cereb. Cortex 17, 2553–2561. 10.1093/cercor/bhl161. 17205974

[24] Ebisch SJH, Perrucci MG, Ferretti A, Del Gratta C, Romani GL, and Gallese V (2008). The sense of touch: embodied simulation in a visuotactile mirroring mechanism for observed animate or inanimate touch. J. Cogn. Neurosci. 20, 1611–1623. 10.1162/jocn.2008.20111. 18345991

[25] Kuehn E, Haggard P, Villringer A, Pleger B, and Sereno MI (2018). Visually-driven maps in area 3b. J. Neurosci 38, 1295–1310. 10.1523/JNEUROSCI.0491-17.2017. 29301873 PMC6596270

[26] Kuehn E, Trampel R, Mueller K, Turner R, and Schütz-Bosbach S (2013). Judging roughness by sight-A 7-tesla fMRI study on responsivity of the primary somatosensory cortex during observed touch of self and others. Hum. Brain Mapp 34, 1882–1895. 10.1002/hbm.22031. 22422484 PMC6869993

[27] Meyer K, Kaplan JT, Essex R, Damasio H, and Damasio A (2011). Seeing touch is correlated with content-specific activity in primary somatosensory cortex. Cereb. Cortex 21, 2113–2121. 10.1093/cercor/bhq289. 21330469 PMC3155604

[28] Pihko E, Nangini C, Jousmäki V, and Hari R (2010). Observing touch activates human primary somatosensory cortex. Eur. J. Neurosci 31, 1836–1843. 10.1111/j.1460-9568.2010.07192.x. 20584188

[29] Schaefer M, Xu B, Flor H, and Cohen LG (2009). Effects of different viewing perspectives on somatosensory activations during observation of touch. Hum. Brain Mapp 30, 2722–2730. 10.1002/hbm.20701. 19172650 PMC6870795

[30] Longo MR, Pernigo S, and Haggard P (2011). Vision of the body modulates processing in primary somatosensory cortex. Neurosci. Lett 489, 159–163. 10.1016/j.neulet.2010.12.007. 21147197

[31] Chan AW-Y, and Baker CI (2015). Seeing is not feeling: posterior parietal but not somatosensory cortex engagement during touch observation. J. Neurosci. 35, 1468–1480. 10.1523/JNEUROSCI.3621-14.2015. 25632124 PMC4308594

[32] Keysers C, Wicker B, Gazzola V, Anton J-L, Fogassi L, and Gallese V (2004). A touching sight: SII/PV activation during the observation and experience of touch. Neuron 42, 335–346. 10.1016/S0896-6273(04)00156-4. 15091347

[33] Morrison I, Lloyd D, Di Pellegrino G, and Roberts N (2004). Vicarious responses to pain in anterior cingulate cortex: is empathy a multisensory issue? Cogn. Cogn. Affect. Behav. Neurosci 4, 270–278. 10.3758/CABN.4.2.270. 15460933

[34] Sharma S, Fiave PA, and Nelissen K (2018). Functional MRI responses to passive, active, and observed touch in somatosensory and insular cortices of the macaque monkey. J. Neurosci 38, 3689–3707. 10.1523/JNEUROSCI.1587-17.2018. 29540550 PMC6705905

[35] Colino FL, Lee J-H, and Binsted G (2017). Availability of vision and tactile gating: vision enhances tactile sensitivity. Exp. Brain Res 235, 341–348. 10.1007/s00221-016-4785-3. 27722789

[36] Haggard P, Christakou A, and Serino A (2007). Viewing the body modulates tactile receptive fields. Exp. Brain Res 180, 187–193. 10.1007/s00221-007-0971-7. 17508208

[37] Kennett S, Taylor-Clarke M, and Haggard P (2001). Noninformative vision improves the spatial resolution of touch in humans. Curr. Biol 11, 1188–1191. 10.1016/S0960-9822(01)00327-X. 11516950

[38] Press C, Taylor-Clarke M, Kennett S, and Haggard P (2004). Visual enhancement of touch in spatial body representation. Exp. Brain Res 154, 238–245. 10.1007/s00221-003-1651-x. 14504860

[39] French B, Chiaro NVD, and Holmes NP (2022). Hand posture, but not vision of the hand, affects tactile spatial resolution in the grating orientation discrimination task. Exp. Brain Res 240, 2715–2723. 10.1007/s00221-022-06450-3. 36074176 PMC9510114

[40] Cardini F, Longo MR, Driver J, and Haggard P (2012). Rapid enhancement of touch from non-informative vision of the hand. Neuropsychologia 50, 1954–1960. 10.1016/j.neuropsychologia.2012.04.020. 22579968 PMC3396851

[41] Cardini F, Longo MR, and Haggard P (2011). Vision of the body modulates somatosensory intracortical inhibition. Cereb. Cortex 21, 2014–2022. 10.1093/cercor/bhq267. 21285259

[42] Deschrijver E, Wiersema JR, and Brass M (2016). The interaction between felt touch and tactile consequences of observed actions: an action-based somatosensory congruency paradigm. Soc. Cogn. Affect. Neurosci 11, 1162–1172. 10.1093/scan/nsv081. 26152577 PMC4927036

[43] Dionne JK, Legon W, and Staines WR (2013). Crossmodal influences on early somatosensory processing: interaction of vision, touch, and task-relevance. Exp. Brain Res 226, 503–512. 10.1007/s00221-013-3462-z. 23455852

[44] Schaefer M, Heinze H-J, and Rotte M (2008). Observing the touched body magnified alters somatosensory homunculus. Neuroreport 19, 901–905. 10.1097/WNR.0b013e328301a629. 18520990

[45] Schaefer M, Flor H, Heinze H-J, and Rotte M (2006). Dynamic modulation of the primary somatosensory cortex during seeing and feeling a touched hand. Neuroimage 29, 587–592. 10.1016/j.neuroimage.2005.07.016. 16099177

[46] Bolognini N, Rossetti A, Maravita A, and Miniussi C (2011). Seeing touch in the somatosensory cortex: a TMS study of the visual perception of touch. Hum. Brain Mapp 32, 2104–2114. 10.1002/hbm.21172. 21305659 PMC6870269

[47] Fiorio M, and Haggard P (2005). Viewing the body prepares the brain for touch: effects of TMS over somatosensory cortex. Eur. J. Neurosci 22, 773–777. 10.1111/j.1460-9568.2005.04267.x. 16101759

[48] Rossetti A, Miniussi C, Maravita A, and Bolognini N (2012). Visual perception of bodily interactions in the primary somatosensory cortex: touch observation in primary somatosensory cortex. Eur. J. Neurosci 36, 2317–2323. 10.1111/j.1460-9568.2012.08137.x. 22626449

[49] Cunningham DA, Machado A, Yue GH, Carey JR, and Plow EB (2013). Functional somatotopy revealed across multiple cortical regions using a model of complex motor task. Brain Res. 1531, 25–36. 10.1016/j.brainres.2013.07.050. 23920009 PMC3931839

[50] Arbuckle SA, Pruszynski JA, and Diedrichsen J (2021). Mapping the integration of sensory information across fingers in human sensorimotor cortex. Neuroscience. 10.1101/2021.07.07.451552. PMC9236287 35606141

[51] Enander JMD, and Jörntell H (2019). Somatosensory cortical neurons decode tactile input patterns and location from both dominant and non-dominant digits. Cell Rep. 26, 3551–3560.e4. 10.1016/j.celrep.2019.02.099. 30917311

[52] Thakur PH, Fitzgerald PJ, and Hsiao SS (2012). Second-order receptive fields reveal multidigit interactions in area 3b of the macaque monkey. J. Neurophysiol 108, 243–262. 10.1152/jn.01022.2010. 22457468 PMC3434610

[53] Qi H-X, Reed JL, Franca JG, Jain N, Kajikawa Y, and Kaas JH (2016). Chronic recordings reveal tactile stimuli can suppress spontaneous activity of neurons in somatosensory cortex of awake and anesthetized primates. J. Neurophysiol 115, 2105–2123. 10.1152/jn.00634.2015. 26912593 PMC4869494

[54] Muret D, Root V, Kieliba P, Clode D, and Makin TR (2022). Beyond body maps: information content of specific body parts is distributed across the somatosensory homunculus. Cell Rep. 38, 110523. 10.1016/j.celrep.2022.110523. 35294887 PMC8938902

[55] Wesselink DB, Sanders Z-B, Edmondson LR, Dempsey-Jones H, Kieliba P, Kikkert S, Themistocleous AC, Emir U, Diedrichsen J, Saal HP, and Makin TR (2022). Malleability of the cortical hand map following a finger nerve block. Sci. Adv 8, eabk2393. 10.1126/sciadv.abk2393. 35452294 PMC9032959

[56] Armenta Salas M, Bashford L, Kellis S, Jafari M, Jo H, Kramer D, Shanfield K, Pejsa K, Lee B, Liu CY, and Andersen RA (2018). Proprioceptive and cutaneous sensations in humans elicited by intracortical microstimulation. Elife 7, e32904. 10.7554/eLife.32904. 29633714 PMC5896877

[57] Kriegeskorte N, Mur M, and Bandettini P (2008). Representational similarity analysis – connecting the branches of systems neuroscience. Front. Syst. Neurosci 2, 4. 10.3389/neuro.06.004.2008. 19104670 PMC2605405

[58] Walther A, Nili H, Ejaz N, Alink A, Kriegeskorte N, and Diedrichsen J (2016). Reliability of dissimilarity measures for multi-voxel pattern analysis. Neuroimage 137, 188–200. 10.1016/j.neuroimage.2015.12.012. 26707889

[59] Nili H, Wingfield C, Walther A, Su L, Marslen-Wilson W, and Kriegeskorte N (2014). A toolbox for representational similarity analysis. PLoS Comput. Biol 10, e1003553. 10.1371/journal.pcbi.1003553. 24743308 PMC3990488

[60] Kikkert S, Pfyffer D, Verling M, Freund P, and Wenderoth N (2021). Finger somatotopy is preserved after tetraplegia but deteriorates over time. Elife 10, e67713. 10.7554/eLife.67713. 34665133 PMC8575460

[61] Makin TR, and Bensmaia SJ (2017). Stability of sensory topographies in adult cortex. Trends Cogn. Sci 21, 195–204. 10.1016/j.tics.2017.01.002. 28214130 PMC6052795

[62] Gazzola V, Spezio ML, Etzel JA, Castelli F, Adolphs R, and Keysers C (2012). Primary somatosensory cortex discriminates affective significance in social touch. Proc. Natl. Acad. Sci. USA 109, E1657–E1666. 10.1073/pnas.1113211109. 22665808 PMC3382530

[63] Zhou Y-D, and Fuster JM (2000). Visuo-tactile cross-modal associations in cortical somatosensory cells. Proc. Natl. Acad. Sci. USA 97, 9777–9782. 10.1073/pnas.97.17.9777. 10944237 PMC16941

[64] Taylor-Clarke M, Kennett S, and Haggard P (2002). Vision modulates somatosensory cortical processing. Curr. Biol 12, 233–236. 10.1016/S0960-9822(01)00681-9. 11839277

[65] Dionne JK, Meehan SK, Legon W, and Staines WR (2010). Crossmodal influences in somatosensory cortex: interaction of vision and touch. Hum. Brain Mapp 31, 14–25. 10.1002/hbm.20841. 19572308 PMC6870919

[66] Eck J, Kaas AL, and Goebel R (2013). Crossmodal interactions of haptic and visual texture information in early sensory cortex. Neuroimage 75, 123–135. 10.1016/j.neuroimage.2013.02.075. 23507388

[67] Kimura T. (2021). Approach of visual stimuli facilitates the prediction of tactile events and suppresses beta band oscillations around the primary somatosensory area. Neuroreport 32, 631–635. 10.1097/WNR.0000000000001643. 33843822 PMC8048733

[68] Gale DJ, Flanagan JR, and Gallivan JP (2021). Human somatosensory cortex is modulated during motor planning. J. Neurosci 41, 5909–5922, JN-RM-0342-21. 10.1523/JNEUROSCI.0342-21.2021. 34035139 PMC8265805

[69] Ariani G, Pruszynski JA, and Diedrichsen J (2022). Motor planning brings human primary somatosensory cortex into action-specific preparatory states. Elife 11, e69517. 10.7554/eLife.69517. 35018886 PMC8786310

[70] Bashford L, Rosenthal I, Kellis S, Pejsa K, Kramer D, Lee B, Liu C, and Andersen RA (2021). The neurophysiological representation of imagined somatosensory percepts in human cortex. J. Neurosci 41, 2177–2185. 10.1523/JNEUROSCI.2460-20.2021. 33483431 PMC8018772

[71] Yoo S-S, Freeman DK, McCarthy JJ, and Jolesz FA (2003). Neural substrates of tactile imagery: a functional MRI study. Neuroreport 14, 581–585. 10.1097/00001756-200303240-00011. 12657890

[72] Christie BP, Charkhkar H, Shell CE, Marasco PD, Tyler DJ, and Triolo RJ (2019). Visual inputs and postural manipulations affect the location of somatosensory percepts elicited by electrical stimulation. Sci. Rep 9, 11699. 10.1038/s41598-019-47867-1. 31406122 PMC6690924

[73] Flesher SN, Collinger JL, Foldes ST, Weiss JM, Downey JE, Tyler-Kabara EC, Bensmaia SJ, Schwartz AB, Boninger ML, and Gaunt RA (2016). Intracortical microstimulation of human somatosensory cortex. Sci. Transl. Med 8, 361ra141. 10.1126/sci-translmed.aaf8083. 27738096

[74] Pandarinath C, and Bensmaia SJ (2022). The science and engineering behind sensitized brain-controlled bionic hands. Physiol. Rev 102, 551–604. 10.1152/physrev.00034.2020. 34541898 PMC8742729

[75] Gonzalez-Franco M, Ofek E, Pan Y, Antley A, Steed A, Spanlang B, Maselli A, Banakou D, Pelechano N, Orts-Escolano S, (2020). The Rocketbox library and the utility of freely available rigged avatars. Front. Virtual Real 1, 561558. 10.3389/frvir.2020.561558.

[76] Christie BP, Tat DM, Irwin ZT, Gilja V, Nuyujukian P, Foster JD, Ryu SI, Shenoy KV, Thompson DE, and Chestek CA (2015). Comparison of spike sorting and thresholding of voltage waveforms for intracortical brain-machine interface performance. J. Neural. Eng 12, 016009. 10.1088/1741-2560/12/1/016009. 25504690 PMC4332592

[77] Dai J, Zhang P, Sun H, Qiao X, Zhao Y, Ma J, Li S, Zhou J, and Wang C (2019). Reliability of motor and sensory neural decoding by threshold crossings for intracortical brain-machine interface. J. Neural. Eng 16, 036011. 10.1088/1741-2552/ab0bfb. 30822756

[78] Diedrichsen J, Berlot E, Mur M, Schutt HH, Shahbazi M, and Kriegeskorte N (2021). Comparing representational geometries using whitened unbiased-distance-matrix similarity. Preprint at ArXiv. 10.48550/arXiv.2007.02789.

